# The effects of microdose LSD on time perception: a randomised, double-blind, placebo-controlled trial

**DOI:** 10.1007/s00213-018-5119-x

**Published:** 2018-11-26

**Authors:** Steliana Yanakieva, Naya Polychroni, Neiloufar Family, Luke T. J. Williams, David P. Luke, Devin B. Terhune

**Affiliations:** 1 Department of Psychology, Goldsmiths, University of London, 8 Lewisham Way, New Cross, London, SE14 6NW UK; 2 Eleusis Pharmaceuticals Ltd, London, UK; 3 Centre for Psychiatry, Division of Brain Sciences, Imperial College London, London, UK; 4 Department of Psychology, Social Work, & Counselling, University of Greenwich, London, UK; 5 Department of Experimental Psychology, University of Oxford, Oxford, UK

**Keywords:** Interval timing, LSD, Microdosing, Older adults, Striatum

## Abstract

**Rationale:**

Previous research demonstrating that lysergic acid diethylamide (LSD) produces alterations in time perception has implications for its impact on conscious states and a range of psychological functions that necessitate precise interval timing. However, interpretation of this research is hindered by methodological limitations and an inability to dissociate direct neurochemical effects on interval timing from indirect effects attributable to altered states of consciousness.

**Methods:**

We conducted a randomised, double-blind, placebo-controlled study contrasting oral administration of placebo with three microdoses of LSD (5, 10, and 20 μg) in older adults. Subjective drug effects were regularly recorded and interval timing was assessed using a temporal reproduction task spanning subsecond and suprasecond intervals.

**Results:**

LSD conditions were not associated with any robust changes in self-report indices of perception, mentation, or concentration. LSD reliably produced over-reproduction of temporal intervals of 2000 ms and longer with these effects most pronounced in the 10 μg dose condition. Hierarchical regression analyses indicated that LSD-mediated over-reproduction was independent of marginal differences in self-reported drug effects across conditions.

**Conclusions:**

These results suggest that microdose LSD produces temporal dilation of suprasecond intervals in the absence of subjective alterations of consciousness.

## Introduction

Our perception of time is important for momentary updating and integration of perceptual information in working memory and is thereby increasingly being recognised as an integral feature of consciousness (Wittmann 2015; Yin et al. 2016). In turn, distortions in *interval timing* (time perception in the milliseconds to minutes range) are a hallmark feature of altered states of consciousness (Berkovich-Ohana and Wittmann 2017; Preller and Vollenweider 2016; Wittmann et al. 2014), as well as psychiatric disorders characterised by disruptions of consciousness, such as schizophrenia and the dissociative disorders (Allman and Meck 2012; Giersch et al. 2015; Simeon et al. 2007; Spiegel et al. 2013).

A striking instance of the close coupling of consciousness and interval timing is observed under lysergic acid diethylamide (LSD). As part of a broad set of alterations in different dimensions of consciousness (Preller and Vollenweider 2016), such as declines in self-related processing and other changes in perception, LSD is associated with both subjective distortions in time perception (DeShon et al. 1952; Kenna and Sedman 1964; Liechti et al. 2016; Savage 1955) and changes in performance on behavioural measures of interval timing (Aronson et al. 1959; Boardman et al. 1957) (for a review see Preller and Vollenweider 2016). For example, LSD (1–2 μg/kg) has been shown to produce a tendency to underestimate the duration of long suprasecond intervals (15–240 min; Aronson et al. 1959) or to increase variability in interval timing for 1 min intervals (Boardman et al. 1957) (although the latter finding has not been replicated [Aronson et al. 1959; Wittmann et al. 2007]). One of the most methodologically rigorous studies to date in this domain observed that the serotonin agonist psilocybin, which has similar characteristics to LSD (Nichols 2016), produced under-reproduction of long suprasecond intervals (4000–5000 ms, but not 1500–2500 ms) (Wittmann et al. 2007). This result implicates serotonin in suprasecond human interval timing (see also Rammsayer 1989; Wackermann et al. 2008), potentially through 5-HT_2A_-mediated inhibition of dopamine (De Gregorio et al. 2016), which is believed to play an important mechanistic role in the perception of time (Allman and Meck 2012; Coull et al. 2011; Matell and Meck 2004; Rammsayer 1999; Soares et al. 2016; Terhune et al. 2016b; Vatakis and Allman 2015; Wiener et al. 2011) (for a review, see Coull et al. 2011). Given the role of interval timing across a range of psychological functions (Allman et al. 2014; Matthews and Meck 2016; Merchant et al. 2013), distorted timing under LSD may contribute to, or underlie, broader cognitive and perceptual effects of this drug. Therefore, elucidating its impact on interval timing is likely to inform neurochemical models of interval timing as well as our broader understanding of the effects of LSD on cognition and perception.

Although multiple studies have reported that LSD produces distortions in time perception as indexed by subjective reports, the small number of studies that used behavioural tasks (Aronson et al. 1959; Boardman et al. 1957) possessed one or more methodological limitations including the absence of placebo controls, randomisation, and double-blind protocols, and small sample sizes and number of experimental trials. A further as of yet unaddressed issue has the potential to directly inform the neurochemical basis of distorted timing. Previous research has been unable to determine whether distorted timing under LSD is attributable to the neurochemical impact of LSD on the neurophysiological substrates of interval timing (Coull et al. 2011) *or* the induction of an altered state of consciousness per se (Liechti et al. 2016; Nichols 2004; Preller and Vollenweider 2016). The administration of LSD doses that are only barely perceptible (< 20 μg; *microdosing*) produces only minor changes in perception and cognition (Greiner et al. 1958) and thereby offers the possibility of partially dissociating the direct, albeit attenuated, neurochemical impact of LSD on interval timing subsystems from its indirect effects on timing through the modulation of conscious states. Indeed, previous research has shown that interval timing tasks may be more sensitive to the effects of psychedelics relative to other cognitive (e.g., working memory) tasks (Wittmann et al. 2007). Accordingly, behavioural measures of interval timing might be especially well-suited to study the psychological effects of microdoses of LSD (see also Wackermann et al. 2008).

The present study used a randomised, double-blind, placebo-controlled design to explore the effects of microdose LSD on time perception. Older adults were randomly allocated to a placebo condition or one of the three LSD microdoses (5, 10, and 20 μg) and completed a temporal reproduction task spanning subsecond and suprasecond intervals (800–4000 ms) approximately 3 h post-dosing. To determine whether any impact of LSD on interval timing converged with peak times of self-perceived drug effects and the potential induction of an altered state of consciousness, participants regularly completed self-report measures of different subjective drug effects. Insofar as there is substantial evidence that LSD alters psychological functions by targeting 5-HT_2A_ receptors (Halberstadt 2015; López-Giménez and González-Maeso 2018; Nichols 2016; Preller et al. 2017), one possible outcome was that microdoses of LSD would produce temporal under-reproduction, as observed with psychoactive doses of psilocybin in this same interval range (Wittmann et al. 2007) and with LSD for longer intervals (Aronson et al. 1959). By contrast, preliminary non-human animal research suggests that LSD might have biphasic effects in which it functions as a serotonin agonist during an early phase and as a dopamine agonist at a later phase (Freedman 1984; Marona-Lewicka and Nichols 2007; Marona-Lewicka et al. 2005; Watts et al. 1995) (for reviews, see De Gregorio et al. 2016; Nichols 2016). These effects have not yet been replicated in human studies or microdoses, to our knowledge, but if this biphasic effect generalises to the present context, microdose LSD after 3 h post-dosing might be expected to produce temporal over-reproduction, as observed with dopamine agonists (Coull et al. 2011).

## Methods

### Participants

Forty-eight healthy native English-speaking older adults (44% female; 56% male) aged between 55 and 75 years of age (*M =* 62.92, *SD* = 5.65) took part in the clinical trial. Participants were recruited at the Early Phase Clinical Unit, Northwick Park Hospital, UK, and were compensated for travel expenses and for participating in the study in accordance with ethical approval. All participants met the following inclusion criteria: (1) no experience of LSD use within the preceding 5 years; (2) female participants were postmenopausal; and (3) male participants with a female partner agreed to the double barrier method of contraception and to not donate sperm for 3 months after the last dose. They also did not meet the following exclusion criteria: (1) history of psychiatric, respiratory, gastrointestinal, renal, hepatic, haematological, lymphatic, neurological, cardiovascular, musculoskeletal, genitourinary, immunological, dermatological, connective tissue or sleep diseases or disorders, and/or intracranial hypertension, impaired consciousness, lethargy, and brain tumour, atopy, hypersensitivity, skin allergies, or allergic reactions to drugs; (2) resting blood pressure, exceeding 160 mmHg (systolic) and 90 mmHg (diastolic), averaged across four assessments taken on the screening day, laboratory test results outside the reference ranges; and/or positive results for hepatitis B surface antigen (HBsAg), hepatitis C virus, or human immunodeficiency virus; (3) current smokers or history of drug abuse or dependence in the last 12 months or positive drug results and alcohol test at screening; (4) use of any prescription drugs or over-the-counter medication therapy, including mega dose vitamin therapy, within 7 days of the first dosing (unless agreed as non-clinically relevant by an investigator and the medical monitor), or receipt of chronic administration of tricyclic antidepressant or lithium or acute administration of selective serotonin reuptake inhibitors, haloperidol, serotonin-norepinephrine reuptake inhibitors, monoamine oxidase inhibitor, over-the-counter doses of 5-HT or St. John’s Wort, or ayahuasca; (5) received or donated blood within the 3 months prior to the first dose; (6) lifetime presence of non-drug-induced psychotic symptoms, a first or second degree relative with psychotic disorders; presence of manic or hypomanic episode, major depressive episode, or lifetime substance abuse in the past 5 years; (7) history of cataract, glaucoma, or any other ophthalmic conditions, hearing loss of more than 40 dB, or veins unsuitable for venepuncture and/or cannulation; and (8) inability to use a computer to the required minimum level.

### Experimental protocol

This study was part of a larger clinical trial evaluating the safety and tolerability of microdoses of LSD in healthy participants. The trial adopted a randomised, double-blind, placebo-controlled design. Participants were randomly assigned to one of the four cohorts (*n* = 12 per cohort) that received different administrations of placebo or LSD (placebo [distilled water], 5, 10, and 20 μg LSD). The study was conducted in compliance with the study protocol and the ethical principles of the Declaration of Helsinki. The study protocol and informed consent form were approved by the Independent Ethics Committee for the Early Phase unit in accordance with the International Conference on Harmonisation, Good Clinical Practice, and the UK law.

The manufacturer of the drug product was Onyx Scientific Limited UK, to cGMP standards. Onyx Scientific Limited UK also undertook the pharmaceutical form development work to establish the optimal process for the manufacturer of the three doses of LSD. The doses were made by the pharmacy at the clinical trial facility under their manufacturing licence conditions and following the process outlined in the IMPD. A manual containing detailed instructions for the storage, manufacture of the doses, dispensing, and reconciliation was followed and worksheets were monitored by an independent clinical research associate during the study. LSD tartrate was prepared as a solution in distilled water. Placebo was distilled water only (indistinguishable from the LSD solution). Stability testing was done for a 6-month period.

### Materials

#### Temporal reproduction task

In each condition, participants completed a temporal reproduction task (Wittmann et al. 2007). In this task, participants had to estimate and memorise the duration of a blue circle and then hold down the space bar for the same perceived duration. Each trial consisted of a brief cue (“memorise”; 750 ms), a blank jittered inter-stimulus interval (425–650 ms), and a target stimulus interval (blue circle [80 × 80 pixels] on a canvas of 1280 × 800; approximately 2 cm in diameter) of varying duration (800, 1200, 1600, 2000, 2400, 2800, 3200, 3600, or 4000 ms). The stimulus was proceeded by a second blank inter-stimulus interval (500 ms), followed by a response cue (“reproduce”), which prompted participants to respond by reproducing the entire stimulus interval with a motor response. Upon depressing the space bar to initiate the reproduction interval, a blue circle was presented again and remained on the monitor until the spacebar was released. Subsequently, participants were presented with a blank inter-trial interval (500 ms) before proceeding to the next trial.

#### Subjective Drug Effects Visual Analogue Scale

The Subjective Drug Effects Visual Analogue Scale (SDEVAS) consisted of 22 questions drawn from different sources (*Drug Effects Questionnaire*; Shram et al. 2011; *Addiction Research Centre Inventory*; Haertzen et al. 1963; *Subjective Effects of Substances with Abuse Potential*; Farré et al. 2007). These data will be reported separately; for the purposes of the current study, we report descriptive data for the following five questions: (1) “Do you feel a drug effect?”; (2) “Do you feel high?”; (3) “Do your surroundings appear different or changed?”; (4) “Do you feel your ability to concentrate is the same, better, or worse than normal?”; and (5) “Are you experiencing unusual thoughts?” Participants responded to each question using a VAS with scores ranging from 0 to 100.

### Procedure

The clinical trial took place in an inpatient setting. Signed, written informed consent was obtained during the screening period prior to the initiation of any clinical screening procedures. During the informed consent process, the purpose of the study, the procedure, and potential hazards were explained to the participants. Participants were screened up to 28 days prior to first dosing. The screening included an assessment of medical history, demographics (gender, age, ethnicity, use of alcohol, and tobacco), medication use, psychiatric history, and a physical examination (weight, height, blood pressure, pulse rate, 12-lead ECG, hearing test, urine analysis, urine drug test, serology, haematology, and serum chemistry). Adverse events were monitored throughout the trial.

Each participant received six single doses of their assigned treatment every 3 days. On dosing days, participants were administered the treatment just prior to (within 20 min of) a standardised breakfast. Participants were not permitted to consume caffeine on dosing days but there were no other restrictions on the consumption of food or beverages during the study. Breakfast was served following 10 h of fasting and participants were served a standardised lunch 4 h after dosing. Fluids were restricted for 1 h before and 1 h after dosing. Participants were not allowed to leave the ward within the first 60 min post-dose. Lunch was provided 4 h post-dose. On dosing days, participants completed the SDEVAS pre-dose and then 30, 60, 90, 120, 150, 180, 240, 300, 360, and 420 min post-dose. The scale took approximately 5 min to complete.

On each dosing day, participants completed a battery of cognitive and perceptual tasks, which will be described elsewhere. The *temporal reproduction task* was completed at variable times post-dose on the fourth dosing day for practical reasons. Experimenters were masked to drug condition and dose and unaware of the hypotheses. Participants completed the task whilst sitting in their beds using a 17-in. laptop (Lenovo; G770) that was positioned on an overbed table at a distance of approximately 60 cm. Stimulus presentation was implemented using Psytools 1.39 (Psytools, Delosis, London, UK). Instructions were provided on-screen and by experimenters. Participants completed one practice block of nine randomised trials and four experimental blocks of 27 randomised trials, amounting to 108 trials (12 per interval). The task took approximately 15–20 min to complete. The participants were released on each dosing day once all tasks had been completed and following a satisfactory subject release interview administered by a psychiatrist. Participants also attended a 4-week follow-up session.

### Statistical analyses

The dependent variables in this study include self-report SDEVAS scores, task completion time, and three performance indices in the temporal reproduction task. Other than those described below, there were no outliers. SDEVAS data were missing for eight participants (*n* = 2 in each dose condition). Analyses of between-group differences in demographic variables and task completion times were performed with chi-squared tests and one-way analyses of variance (ANOVAs). Welch ANOVAs were used for all between-group data that violated the assumption of homogeneity of variance. Analyses were performed in MATLAB (v. 2017b, Mathworks, Inc., Natick, MA) and SPSS (v. 22, IBM, Armonk, NY).

**Fig. 1:**
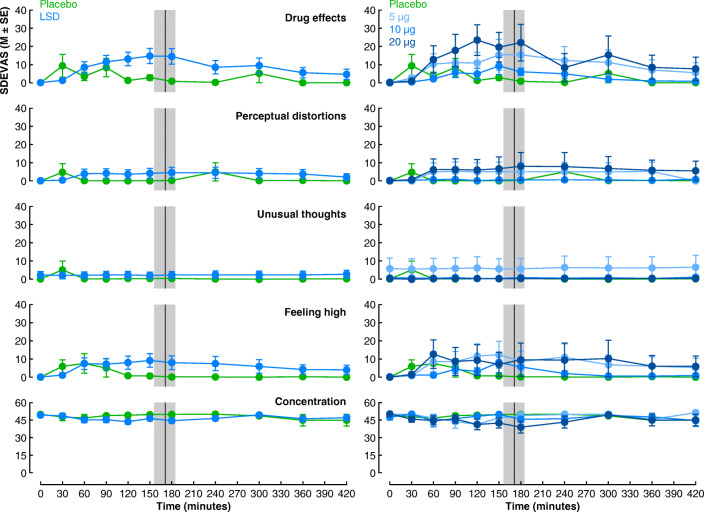
Self-reports on *Subjective Drug Effects Visual Analogue Scales* (SDEVAS) as a function of time (minutes) post-dosing and drug (placebo vs. LSD) (left) and dose (placebo vs. 5 vs. 10 vs. 20 μg) (right) (see the “Methods” section for exact questions). The black line and grey region denote the mean task completion time and 95% CIs (10,000 bootstrap resamples), respectively

SDEVAS scores were analysed in two separate sets of mixed-model ANOVAs: the first treated drug as a between-group independent variable (placebo vs. LSD) whereas the second included dose as a between-group independent variable (placebo vs. 5 vs. 10 vs. 20 μg LSD). Each set of ANOVAs included interval as a repeated measures independent variable (800 vs. 1200 vs. 1600 vs. 2000 vs. 2400 vs. 2800 vs. 3200 vs. 3600 vs. 4000 ms). We report *η*^2^ and *η*_*p*_^2^ as measures of effect size. The assumption of sphericity was violated in most analyses, and thus, a Greenhouse-Geisser correction was applied (uncorrected *df*s are reported). Insofar as we observed suggestive between-group differences in SDEVAS that did not achieve statistical significance, we performed exploratory analyses (*α* < .01) to determine whether participants differed in SDEVAS measures at 180 min, the time point when SDEVAS scores were measured that was closest to the task completion time (Fig. 1).

The two principal dependent variables in the *temporal reproduction task* were median reproduction time and coefficient of variation (CV; *SD*/*Mdn*), an index of response variability, for each stimulus interval. Screening of the median reproduction times revealed two extreme outliers (< *M –* 3.5 *SD*s). Both participants (one each from the 5 and 20 μg conditions) displayed similar, atypically fast, reproduction times (ranges: 160–250 ms) uniformly across all stimulus intervals, suggesting a failure to understand, or an inability to perform, the task. These data were excluded from the analyses. Median reproduction times and the CVs were winsorised using the 97.5 and 2.5 percentiles (*μ* + *σ* × z, where *z* = 1.96 [max]; *z* = − 1.96 [min]). These data were analysed as above using mixed-model ANOVAs.

The third dependent variable in the temporal reproduction data involved analysing the extent to which reproduction times *increased* with longer stimulus intervals. This provides a measure of the magnitude of change in over-reproduction with longer intervals and was computed by performing within-participant regression analyses in which reproduction times (for all trials) were regressed on stimulus intervals within each participant. We subsequently contrasted the resultant beta coefficients, which provide an index of the slope of the change in reproduction times (higher values reflect a steeper slope), using one-way between-group ANOVAs.

A final set of exploratory analyses sought to determine whether condition-specific differences in temporal reproduction were independent of suggestive differential self-reported drug effects across conditions. Toward this end, we performed two sets of two-stage hierarchical regression analyses that alternately included drug or dose as predictors. Median reproduction times in each stimulus interval were regressed on self-reported drug effects (SDEVAS) in the first stage with drug (0 = placebo, 1 = LSD) or dose (0 = placebo, 1 = 5 μg, 2 = 10 μg, 3 = 20 μg LSD) condition added to the model in the second stage. We report *R*^2^ (first block) and ∆*R*^2^ (second block), and corresponding *p*s for each stage, and interpret significant model improvements (∆*R*^2^) as an indication that drug or dose predicts individual differences in temporal reproduction independently of self-reported drug effects.

## Results

### Sample demographics

The sample demographics for the different conditions are presented in Table 1. Participants in the different groups did not differ in gender distributions, *χ*^2^_3_ = 2.29, *p* = .52, Cramer’s *V* = .22, or age, *F*_3,47_ = .29, *p* = .83, *η*^2^ = .02. Participants completed the temporal reproduction task at variable time points post-dosing (*M* = 170 min; CIs 156, 183) (Fig. 1). Nevertheless, there were no significant differences in task completion times (time post-dose at which the task was completed) across drugs (placebo: *M* = 171, *SD* = 40; LSD: *M* = 172, *SD* = 50), *F*_1,24.26_ < .01, *p* = .95, *η*^2^s < .01, or doses (placebo: *M* = 171, *SD* = 40; LSD 5 μg: *M* = 175, *SD* = 58; LSD 10 μg: *M* = 158, *SD* = 46; LSD 20 μg: *M* = 184, *SD* = 47), *F*_1,22.91_ = .58, *p* = .64, *η*^2^s = .03. These results suggest that the task was completed at a relatively uniform post-dose time across conditions.

**Table 1:** Demographic information for participants as a function of drug condition

Observation	Placebo (*n* = 12)	LSD 5 μg (*n* = 12)	LSD 10 μg (*n* = 12)	LSD 20 μg (*n* = 12)	Total (*N* = 48)
Gender [female:male]	6:6	6:6	3:9	6:6	21:27
Age [*M* (*SD*)]	63.50 (6.29)	63.17 (4.80)	61.58 (6.64)	63.42 (5.2)	62.92 (5.65)

### Subjective drug effects (SDEVAS)

We analysed five self-report measures concerning subjective changes in cognition and perception (see Fig. 1) with two sets of mixed model ANOVAs with (post-dose) time as a repeated measures independent variable and drug or dose as between-group independent variables, respectively. In addition to two participants who were excluded because of outlying performance in the temporal reproduction task, six participants were excluded because of missing data at one or more time points. Although there were numerical tendencies for larger reported *drug effects* in the LSD condition, there were no significant main effects of drug, *F*s < 1.38, *p*s > .25, *η*_*p*_^2^s < .04, or time, *F*s < 2.26, *p*s > .06, *η*_*p*_^2^s < .06, or time × drug interactions, *F*s < 2.4, *p*s > .05, *η*_*p*_^2^s < .07, on any of the measures. Similarly, there were no significant main effects of dose, *Fs* < 1.34, *ps* > .28, *η*_*p*_^2^ s < .10, or time, *F*s < 3.3, *p*s > .05, *η*_*p*_^2^s < .22, except on *drug effects*, *F*_10,360_ = 4.91, *p* < .001, *η*_*p*_^2^ = .12, which reflected a tendency for reports to increase from baseline to 120–180 min and subsequently decline again, and no significant time × dose interactions, *F*s < 1.5, *p*s > .18, *η*_*p*_^2^s < .12. Insofar as there appeared to be numerical differences across conditions in SDEVAS scores that overlapped with the task completion time, particularly for *drug effects* and *feeling high*, we performed exploratory condition contrasts (*α* < .01) on SDEVAS ratings at 180 min post-dosage. There was a suggestive main effect of drug on drug effects, *F*_1,38_ = 5.18, *p* = .030, *η*^2^ = .04, but no significant effects for any of the other SDEVAS scores, *F*s < 3.6, *p*s > .06, *η*^2^s < .04. Similarly, there were no significant effects of dose on any of the SDEVAS scores, *F*s < 2.3, *p*s > .12, *η*^2^s < .07. Cumulatively, these results suggest that microdose LSD did not produce robust changes in a range of conscious states, as indexed by self-report measures.

### Drug-specific effects

#### Reproduction times

**Fig. 2:**
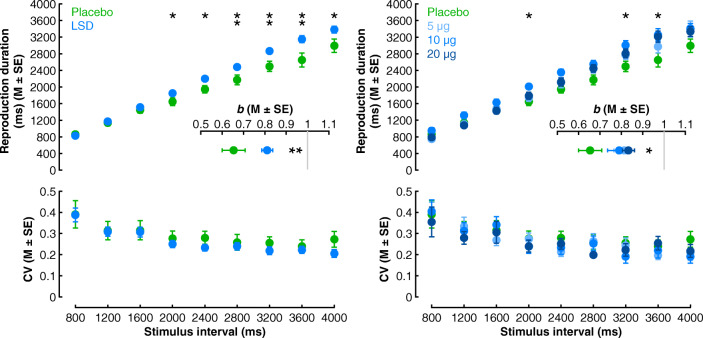
Temporal reproduction performance as a function of drug (placebo vs. LSD) (left) and dose (placebo vs. 5 vs. 10 vs. 20 μg) (right). Reproduction durations (top), beta coefficients from within-participant regression analyses of reproduction durations on stimulus intervals (insets), and reproduction variability (coefficient of variation; CV) (bottom). *p < .05, **p < .01

Reproduction times in the temporal reproduction task are presented in Fig. 2 as a function of drug. There was a main effect of interval, *F*_8,352_ = 411.63, *p* < .001, *η*_p_^2^ = .90, reflecting longer reproduction times as the stimulus interval increased. A main effect of drug was also observed, *F*_1,44_ = 6.18, *p* = .017, *η*_p_^2^ = .12, with participants displaying longer reproduction times in the LSD, relative to the placebo, condition. These effects were further mediated by a drug × interval interaction, *F*_8,352_ = 5.13, *p* = .003, *η*_p_^2^ = .10. Subsidiary analyses (*α* < .01) revealed drug effects on reproduction times at three intervals, with relatively uniform effect sizes (~ .16): 2800 ms: *F*_1,45_ = 7.53, *p* = .009, *η*^2^ = .15; 3200 ms: *F*_1,45_ = 8.34, *p* = .006, *η*^2^ = .16; and 3600 ms: *F*_1,45_ = 8.47, *p* = .006, *η*^2^ = .16. Main effects of drug were also suggestive (*α* < .05) for the 2000, 2400, and 4000 ms intervals with comparable effect sizes, *F*s < 5.38, *p*s < .04, *η*^2^s > .11, whereas they were reliably non-significant, with negligible effect sizes, for the 800–1600 ms intervals, *F*s < .59, *p*s > .70, *η*^2^s < .01.

In order to determine whether temporal over-reproduction in the LSD condition was independent of potential differential self-reported drug effects across conditions, we next performed a hierarchical regression on reproduction times in each interval separately with drug effect ratings at 180 min post-dosage included in the first step as a nuisance variable and drug condition in the second step. Drug effects did not significantly predict reproduction times for any of the stimulus intervals in the first step, *R*^2^s < .06, *p*s > .17, although there was a weak trend toward significance in the 2800 ms interval condition, *R*^2^ = .09, *p* = .064. In the second step, drug condition did not improve the models for 800–1600 ms intervals, ∆*R*^2^s < .06, *p*s > .18, but reliably significantly improved the models for all other intervals (2000–4000 ms), ∆*R*^2^ range .11–.20, *p* range .025–.002. In all cases, the LSD condition was positively associated with reproduction times. These results corroborate the foregoing ANOVAs and indicate that differences in temporal reproduction across drug conditions are independent of potential differential subjective drug effects across conditions.

#### Reproduction slopes

To further clarify whether LSD was associated with steeper reproduction slopes, we performed within-participant regression analyses on participants’ individual reproduction data with stimulus interval as a predictor and subsequently contrasted beta coefficients across conditions. The analyses revealed a main effect of drug, *F*_1,44_ = 8.53, *p* = .005, *η*^2^ = .16, reflecting steeper slopes in the LSD condition, which further corroborates the tendency for temporal over-production to be more pronounced selectively at longer stimulus intervals (Fig. 2).

#### Reproduction variability

Reproduction variability data, as indexed by coefficients of variation (CVs), are presented in Fig. 2. There was a main effect of interval, *F*_8,352_ = 11.41, *p* < .001, *η*_p_^2^ = .21, reflecting a reduction in variability as the stimulus interval increased; however, there were neither significant main effects of drug, *F*_1,44_ = 0.70, *p* = .41, *η*_p_^2^ = .02, nor a drug × interval interaction, *F*_8,352_ = .47, *p* = .81, *η*_*p*_^2^ = .01. This suggests that LSD is not significantly associated with atypical variability in temporal reproduction.

### Dose-specific effects

#### Reproduction times

Analyses of reproduction times as a function of dose (Fig. 2) revealed a main effect of interval, *F*_8,336_ = 584.017, *p* < .001, *η*_*p*_^2^ = .93, as previously observed, and a main effect of dose, *F*_3,42_ = 3.22, *p* = .032, *η*_*p*_^2^ = .19. These effects were further mediated by a dose × interval interaction, *F*_24,336_ = 2.15, *p* = .037, *η*_*p*_^2^ = .13. Subsidiary analyses did not reveal effects of dose on reproduction times at specific intervals (*α* < .01), although there were suggestive effects (*α* < .05) with uniformly strong effect sizes (~ .21) at 2000 ms, *F*_3,45_ = 3.74, *p* = .018, *η*^2^ = .21, 3200 ms, *F*_3,45_ = 3.67, *p* = .019, *η*^2^ = .21, and 3600 ms, *F*_3,45_ = 3.5, *p* = .023, *η*^2^ = .20. In each case, reproduction times were longer in the 10 μg dose condition than in the placebo condition, post hoc Tukey’s *p* range .01–.029. All other dose effects were non-significant, *F*s < 3.17, *p*s > .058, *η*^2^s < .18.

Although self-reported drug effects did not significantly differ across dose conditions at 180 min post-dosage, as in the drug-specific analyses, we performed hierarchical regressions to assess whether the observed dose-specific differences in temporal reproduction were independent of self-reported drug effects. As described above, in the first step, drug effects did not significantly predict reproduction times in any of the stimulus intervals. In the second step, dose did not improve the models for 800–1600 ms or 4000 ms intervals, ∆*R*^2^s < .07, *p*s > .10, but significantly improved the models for all other intervals (2000–3600 ms), ∆*R*^2^ range .11–.20, *p* range .040–.004. In all cases, an increased dose was positively associated with reproduction times. These results suggest a positive linear dosage effect on temporal reproduction that is independent of potential differential subjective drug effects across conditions.

#### Reproduction slopes

A re-analysis of within-participant regression beta coefficients as a function of dose (Fig. 2) revealed a significant main effect of dose, *F*_3,42_ = 2.88, *p* = .047, *η*^2^ = .17. Post hoc Tukey’s tests revealed suggestively larger coefficients in the 20 μg relative to the placebo condition, *p* = .055, but no other significant differences, *p*s > .10. This suggests a weak tendency for over-production in the 20 μg condition to be more pronounced for longer stimulus intervals.

#### Reproduction variability

The re-analysis of CVs as a function of dose (Fig. 2) replicated the main effect of interval, *F*_8,336_ = 16.66, *p* < .001, *η*_*p*_^2^ = .28, but there was no significant effect of dose, *F*_3,42_ = .242, *p* = .87, *η*_*p*_^2^ = .017, nor a dose × interval interaction, *F*_24,336_ = .87, *p* = .6, *η*_*p*_^2^ = .06. These data corroborate the previous results and suggest that LSD does not significantly impact reproduction variability.

## Discussion

Here we show in a placebo-controlled, double-blind, randomised trial with healthy older adults that microdose LSD produces a tendency to over-reproduce suprasecond intervals on a temporal reproduction task. Dose analyses further suggest potential linear effects of dose on temporal reproduction although over-reproduction tended to be most pronounced with a 10 μg dose. Alterations in temporal reproduction were restricted to intervals exceeding 1600 ms, suggesting that this effect may be interval-specific and restricted to suprasecond interval timing. Participants displayed a weak tendency to report greater subjective drug effects in the LSD conditions, hinting that participants were able to detect their assigned condition. However, LSD was not reliably associated with alterations in different self-reported dimensions of consciousness and the differential temporal reproduction performance across conditions was independent of self-reported drug effects. These results expand upon previous research showing that LSD modulates the perception of time (Aronson et al. 1959; Boardman et al. 1957; DeShon et al. 1952; Liechti et al. 2016; Speth et al. 2016) by indicating that LSD-mediating distorted timing can be independent of an altered state of consciousness. Interval timing appears to be particularly sensitive to the effects of psychedelics and thus represents a valuable method for measuring the psychological effects of these drugs (Wackermann et al. 2008; Wittmann et al. 2007).

A notable result of the present study is that the modulation of interval timing by LSD was restricted to intervals between 2000 and 4000 ms. This effect partially converges with the previous observation that the impact of psilocybin on temporal reproduction was specific to 4000–5000 ms intervals (and not 1500–2500 ms), albeit in the converse direction (Wittmann et al. 2007). Taken together, these results suggest that interval timing is most easily influenced by psychedelic drugs when intervals exceed 1600–2500 ms in duration. Multiple lines of research suggest that interval timing of subsecond and suprasecond intervals is subserved by partially distinct psychological and neurophysiological mechanisms (Coull et al. 2012; Hayashi et al. 2014; Rammsayer 1999; Rammsayer and Troche 2014; Wiener et al. 2011), although the approximate interval breakpoint that distinguishes these systems is poorly understood (Grondin 2014; Lewis and Miall 2009). The present results arguably provide further evidence for a dissociation between these putative timing systems, with LSD influencing a suprasecond system potentially through more cognitive dimensions of timing, including attention and working memory, which are recruited to a greater extent for timing in this interval range (Lewis and Miall 2003; Matthews and Meck 2016; Wittmann et al. 2007).

The present results are at odds with previous research that examined the impact of psychedelic drugs, which primarily function as serotonin agonists, on behavioural indices of time perception. As is the case with two previous studies using LSD (Aronson et al. 1959) and psilocybin (Wittmann et al. 2007), we failed to replicate the finding that LSD enhances variability of interval timing (Boardman et al. 1957). However, our primary finding of temporal over-reproduction in the microdose LSD condition for stimulus intervals from 2000 to 4000 ms in this study is inconsistent with the previous observation that LSD produced under-reproduction of temporal intervals (Aronson et al. 1959). Nevertheless, the latter study used substantially longer intervals (> 15 min), only a single trial per interval, and verbal estimates of duration, rendering comparison with the present study difficult. Our results are also discrepant with those of a previous study that found that relative to baseline, psilocybin produced temporal under-reproduction in an interval range that overlapped with the present study (Wittmann et al. 2007). Moreover, hierarchical regression analyses suggested that the magnitude of temporal over-reproduction under LSD covaries partly with dosage. Divergences between our results (temporal over-reproduction) and those of these previous studies (temporal under-reproduction) are plausibly attributable to the use of microdoses and psychedelic doses, respectively. Psychedelic doses of LSD and psilocybin commonly produce pronounced changes in different dimensions of consciousness, such as hallucinatory percepts (Carhart-Harris et al. 2016a; Liechti 2017; Nichols 2016), which are likely to attract attention and divert it away from the passage of time (Buhusi and Meck 2009). Similarly, the experience of elation in response to psychedelics (Carhart-Harris et al. 2016a) might produce a tendency to underestimate or under-reproduce temporal intervals, as is typically observed during positive affective states (Lake et al. 2016). Finally, a decrease in self-related processing in response to psychedelics (Carhart-Harris et al. 2016b; Liechti 2017; Preller and Vollenweider 2016; Tagliazucchi et al. 2016) would also be expected to produce under-reproduction (Wittmann 2013, 2015; Yin et al. 2016). Thus, the observed direction of distorted timing in the present study is arguably consistent with the relative lack of canonical alterations in consciousness and inconsistent with what is typically observed with serotonin agonists (Wittmann et al. 2007).

Insofar as the cognitive, neurochemical, and neurophysiological effects of microdose LSD are largely unknown, the proposal of plausible mechanisms is necessarily speculative and the following proposals should be treated with caution. Interval timing is closely intertwined with attention and working memory (Buhusi and Meck 2009; Gu et al. 2015; Matthews and Meck 2016), and thus, the present results are plausibly driven by changes in these fundamental cognitive systems. It seems unlikely that the current results can be attributed to *poorer* working memory or selective attention, such as an increase in attentional lapses, as such effects would have been expected to produce temporal under-reproduction (Buhusi and Meck 2009; Terhune et al. 2017; Wittmann et al. 2007). An alternative explanation for our results is that microdose LSD *enhanced* selective attention to duration during the task, resulting in a tendency to over-reproduce temporal intervals (Buhusi and Meck 2009; Lake and Meck 2013). Indirect support for this hypothesis comes from a recent survey of microdose users that found that participants reported being more focused on the first day of microdosing (Polito and Stevenson 2018), although this effect declined on subsequent dosing days and it remains unclear whether the self-reported change in attentional focus in the latter study is attributable to a placebo response. Although we are unable to completely discount this interpretation, participants completed the temporal reproduction task on the fourth dosing day and did not report differential concentration under LSD and previous research shows that both phenomenological and behavioural indices of attentional state covary with individual differences in interval timing (Berry et al. 2014; Terhune et al. 2017).

A final explanation for the present results is that temporal over-reproduction was driven by the activation of D_2_ receptors, as suggested by non-human animal research indicating that LSD functions as a dopamine agonist at a late phase (Marona-Lewicka and Nichols 2007; Marona-Lewicka et al. 2005) (see also De Gregorio et al. 2016; Giacomelli et al. 1998; Rickli et al. 2016). This interpretation is consistent with a wealth of evidence implicating dopamine in interval timing (for reviews, see Coull et al. 2011; Matell and Meck 2004) and in particular that dopamine agonists produce overestimation or over-reproduction of temporal intervals (Buhusi and Meck 2007; Lake and Meck 2013; Maricq et al. 1981). Nevertheless, this interpretation remains controversial because these putative biphasic pharmacological effects have not yet been observed in humans with microdoses or psychoactive doses to our knowledge. Non-human animal research does not always translate to humans (Ioannidis 2012), and thus, this interpretation should be treated with caution until corroborative evidence for biphasic effects is observed in humans.

Future research on the effects of micro- and psychoactive doses of psychedelic drugs on interval timing will benefit from more systematically exploring the mediators of these effects, their interval specificity, their time course, and their neurochemical specificity. Further study of the seemingly differential impact of micro- and psychoactive doses on interval timing tasks is necessary to determine whether the apparent converse effects of these doses on timing are replicable and not attributable to an as of yet unknown confound. Such an orientation will also be valuable in understanding how distorted timing relates to broader alterations in affect, cognition, and perception in response to psychedelic doses of LSD (Carhart-Harris et al. 2016a; Liechti 2017; Terhune et al. 2016a). Concurrent measurement of different psychological and physiological parameters that might mediate distorted timing, such as attention, arousal, memory, working memory, and affect (Lake et al. 2016; Matthews and Meck 2016), will enable a more precise understanding of the psychological variables that underlie changes in interval timing in response to LSD and other psychedelics. The latter approach will be especially beneficial when coupled with the measurement of a wide interval range in order to establish the cognitive and perceptual bases for the repeated observation that distorted timing under psychedelics is restricted to suprasecond intervals (Wittmann et al. 2007). The aim to understand distorted timing in response to psychedelics will further benefit from integrating research on psychedelics with that on germane phenomena known to modulate awareness and time perception (Berkovich-Ohana and Wittmann 2017; Lemercier and Terhune 2018; Noreika et al. 2014; Yin et al. 2016). Repeated measurement of interval timing at multiple time points post-dosage will allow for greater insights into whether and how timing changes during different hypothesised phases of LSD (Marona-Lewicka and Nichols 2007; Marona-Lewicka et al. 2005). Similarly, the use of serotonin and dopamine antagonists at different time points post-dose (Preller et al. 2017) will enable a more robust assessment of the role of these neurochemicals in LSD-mediated distorted timing.

Interpretation of the present results must also consider the limitations of our design. Given the methodological challenges of conducting LSD research, this study, like many other human studies in this domain (Liechti 2017), included a small sample size. The analyses were plausibly underpowered, particularly those pertaining to dosage effects, and we observed multiple suggestive, convergent effects with strong effect sizes that may have met our thresholds for statistical significance with a larger sample size. For example, the lack of clear linear dosage effects in the ANOVAs is potentially due to low statistical power, particularly since evidence for linear dosage effects were observed in the hierarchical regression analyses. Indeed, temporal over-reproduction was observed in multiple dosage conditions relative to placebo at trend levels (.05 < *p*s < .10) and thus would have plausibly achieved statistical significance with larger sample sizes in each dose condition. This limitation is perhaps compounded by the use of a between-group design as a within-group design would have afforded greater internal validity and increased the likelihood that the observed effects are attributable to the drug conditions. For this reason, the non-significant dosage effects should be interpreted with caution. Participants in the different conditions did not significantly differ in the self-report measures, but there was a weak tendency for those in the LSD condition to report greater subjective drug effects, but not other subjective effects, than those in the placebo condition. This effect was not observed across drug doses but it is possible that our small sample sizes attenuated our ability to detect such effects. A further limitation of the study is the absence of a baseline condition for the temporal reproduction task. Although the use of random assignment mitigates the negative impact of the absence of baseline data on the internal validity of the study, such data would allow for stronger inferences regarding the effect of LSD on interval timing. A final limitation of this study is that the sample was comprised entirely of older adults. Older adults display temporal contraction on interval timing tasks (Lustig and Meck 2011; Turgeon and Wing 2012) with some constraints (for a review see Turgeon et al. 2016), potentially due to reduced striatal dopamine receptor availability (Allard and Marcusson 1989; Shingai et al. 2014; Zelnik et al. 1986). Accordingly, the observed changes in interval timing are potentially restricted to this population and not generalizable to younger populations.

The present results suggest that microdose LSD produces a tendency to over-reproduce suprasecond temporal intervals in older adults. Additional evidence from self-report measures suggests that the observed effects are unlikely to be attributable to altered states of consciousness. In particular, although there was a tendency for those in the LSD condition to report greater drug effects, the observed temporal over-reproduction effect was independent of self-report drug effects. Further research is required to replicate these results, including their interval specificity, and identify the neurochemical and cognitive mediators of distorted timing under micro- and psychoactive doses of LSD.
